# Prenatal Ultrasound Screening for External Ear Abnormality in the Fetuses

**DOI:** 10.1155/2014/357564

**Published:** 2014-06-23

**Authors:** Jun Wei, Suzhen Ran, Zhengchun Yang, Yun Lin, Jing Tang, Haitao Ran

**Affiliations:** ^1^Department of Ultrasound, the Second Affiliated Hospital of Chongqing Medical University, Chongqing 400013, China; ^2^Chongqing Health Center for Women and Children, , China Chongqing Health Center for Women and Children, Jintang Street 64, Yuzhong, Chongqing 400013, China

## Abstract

*Objectives.* To investigate the best time of examination and section chosen of routine prenatal ultrasound screening for external ear abnormalities and evaluate the feasibility of examining the fetal external ear with ultrasonography.* Methods*. From July 2010 until August 2011, 42118 pregnant women with single fetus during 16–40 weeks of pregnancy were enrolled in the study. Fetal auricles and external auditory canal in the second trimester of pregnancy were evaluated by routine color Doppler ultrasound screening and systematic screening. Ultrasound images of fetal external ears were obtained on transverse-incline view at cervical vertebra level and mandible level and on parasagittal view and coronal view at external ear level.* Results*. Five fetuses had anomalous ears including bilateral malformed auricles with malformed external auditory canal, unilateral deformed external ear, and unilateral microtia. The detection rate of both auricles was negatively correlated with gestational age. Of the 5843 fetuses undergoing a routine ultrasound screening, 5797 (99.21%) had bilateral auricles. Of the 4955 fetuses following systematic screening, all fetuses (100%) had bilateral auricles. The best time for fetal auricles observation with ultrasonography is 20–24 weeks of pregnancy.* Conclusions*. Detection of external ear abnormalities may assist in the diagnosis of chromosomal abnormalities.

## 1. Introduction

Studies have shown that an incidence of 1 : 6000 until 1 : 6830 newborns has been reported for external ear malformations [1–3]. Ear abnormalities are important in the diagnosis of a variety of congenital malformations or syndromes in newborns [4–6]. For example, reduced ear length is the most consistent phenotypic characteristic of neonates for diagnosis of trisomy 21 [7]. Therefore, dedicated examinations on fetal external ear are imperative.

Ultrasound is generally considered to be a reliable noninvasive method for monitoring and assessing fetal growth and well-being as well as for the early diagnosis of specific disorders associated with the pregnancy [8]. Ultrasonography is, moreover, the method of choice for the diagnosis of congenital abnormalities of the fetus [9]. Its diagnostic performance of routine ultrasound examinations performed in the 1980s and 1990s has been described in several publications. Because of improved ultrasound technology offering better resolution and improvement in knowledge and experience of ultrasound examiners, detection rates of fetal malformations may have increased since the 1990s [10].

External ear has a relatively complex structure and shape that is species specific and is remarkably constant in its basic normal shape [11]. Numerous studies have reported the utility of the US in the evaluation of fetal anatomy and abnormalities. In particular, this modality has been helpful in evaluation of facial abnormalities, hand abnormalities, club feet, skeletal dysplasia, and spinal malformations [12–15]. To the best of our knowledge, there are only a few reports on the diagnosis of external ear abnormalities by ultrasonography which mainly focus on auricular deformities indicative of chromosomal abnormalities [4, 16–18]. The aim of our study was to investigate the best time of examination and section chosen of routine prenatal ultrasound screening for fetal auricular abnormalities and evaluate the feasibility of examining the fetal ears with ultrasonography.

## 2. Materials and Methods

The study was approved by the ethics committee of Chongqing Maternal and Child Health Hospital, and written informed consent was obtained from all subjects. Pregnant women referred for routine fetal ultrasound and who fulfilled the eligibility criteria were invited to participate in the study. Between July 2010 and August 2011, 42118 fetuses were scanned and included in the study. The median maternal age was 31.3 years (range 18–45). Gestational age, calculated from the last menstrual period and confirmed by measuring the fetal crown-rump length, was 16–40 (mean 24.5 ± 1.3) weeks.

Inclusion criteria were as follows: (1) women who were scheduled for routine fetal ultrasound examination between weeks 16 and 24 of pregnancy; (2) women referred for determination of gestational age or growth discrepancy reassurance because of a preceding miscarriage, lack of fetal movement, inability to detect a fetal heartbeat, or other miscellaneous reasons.

Transabdominal scan was performed with Phillips HD11XE (Phillips, U.S.A.), GE Voluson 730 or GE Voluson 730 Expert (GE, Austria) equipment, using both 3.5 and 5.0 MHz transducer. Scans were performed by sonographers or physicians and interpreted by fetal-imaging specialists with comparable experience. The content of routine color Doppler ultrasonography mainly included the measurement of fetal biological indicators in the second and third trimester and basic morphological examination with a focus on the morphstructure of vital organs and screening for lethal or severe abnormalities. In addition, data on any obvious fetal abnormalities were recorded and demographic details and the findings of the ultrasound examinations were entered into a computer database at the time of scan.

Systematic screening was carried out aiming at the high risk pregnancy and doubtful fetal abnormalities in the second trimester observed in the routine ultrasound examination, which was assessed by the Fetal Medicine Foundation, United Kingdom.

Based on the results of routine ultrasound screening and systematic screening for fetus, detailed examination of fetal auricular anatomy was carried out at 16–40 weeks of gestation. In order to observe the bilateral auricles and external auditory canal of fetuses, the scan images of fetal external ear in different sections included transverse-incline view at cervical vertebra level (section A) and mandible level (section B) and parasagittal view (section C) and coronal view (section D) at external ear level [19]. In all cases the ear length measurement was performed and the measurement of ear length was obtained between two points, from the apical part of the helix to the caudal part of the earlobe, with techniques described in previous study by Hatanaka et al. [4].

### 2.1. Statistical Analysis

Numerical data were expressed as median and range. Qualitative data were expressed as percentage. Correlation analysis was used to examine the relation between qualitative variables. *P* < 0.05 was considered as significant. All analyses were performed using SPSS 13.0 software (SPSS, Chicago, IL, USA). By comparing the prenatal diagnosis (presence or absence of anomalies) with the postnatal diagnosis, the latter being taken as a gold standard, we categorized all ultrasound examinations as true positive in cases when a suspected anomaly was confirmed, false positive when a suspected anomaly could not be confirmed, false negative when an anomaly was missed by ultrasound examination, or true negative when no anomalies were present. The sensitivity, specificity, positive predictive value, and negative predictive value were calculated with corresponding exact 95% binomial CIs. In addition, the prevalence of congenital malformations (the proportion of infants affected) was calculated [20].

## 3. Results

Ultrasound scanning of normal fetal auricles between 16 and 40 weeks' gestational age displayed clear and bright-field C- or S-shaped images with hyperecho. Scanning images of parasagittal view and coronal view at external ear level (section C and D) had a higher detection rate which can be used as standard observation sections for fetal external ear. The auricular positions between two auricles can be judged by the scanning images of sections A and B, but the imaging results were more affected by changes of fetal position, placenta, and amniotic fluid. Therefore, these two sections can be used as supplementary for section C and section D.

Of the 42118 fetuses that were screened, 5843 underwent a routine ultrasound examination and 4955 fetuses underwent systematic. Of the 5843 fetuses undergoing a routine ultrasound screening from 16 weeks' gestational age to the birth, 5797 (99.21%) between 16 and 40 weeks' gestational age had bilateral auricles. Of the 4955 fetuses following systematic screening in the second trimester of pregnancy, all fetuses (100%) had bilateral auricles. The detection rate of bilateral ears was negatively correlated with gestational age (*r* = −0.911, *P* < 0.01) and was highest between 20 to 24 weeks of gestational age.  Table 1 shows the distribution of the duration of pregnancy at which the ultrasound examinations were performed.

**Figure 1:**
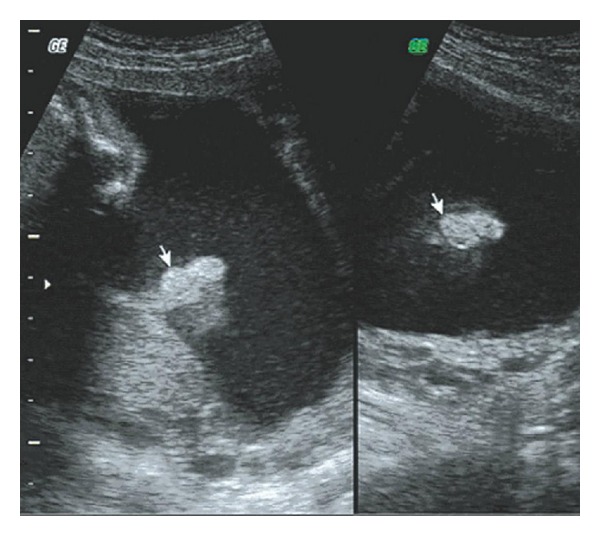
Sonogram of bilateral microtia on auricular parasagittal section.

**Figure 2:**
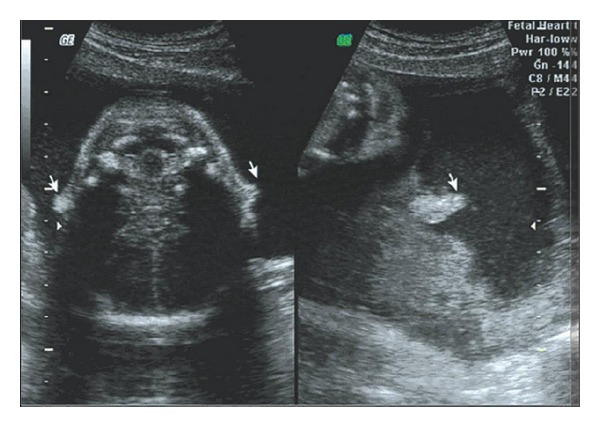
Sonogram of bilateral microtia on retrocolic transverse section.

**Figure 3:**
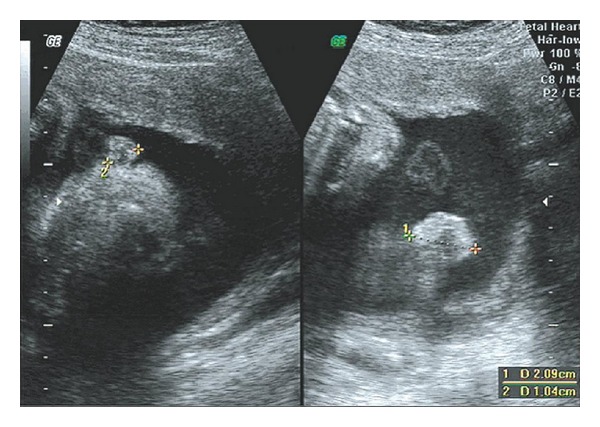
Sonogram of unilateral microtia on auricular parasagittal section.

**Figure 4:**
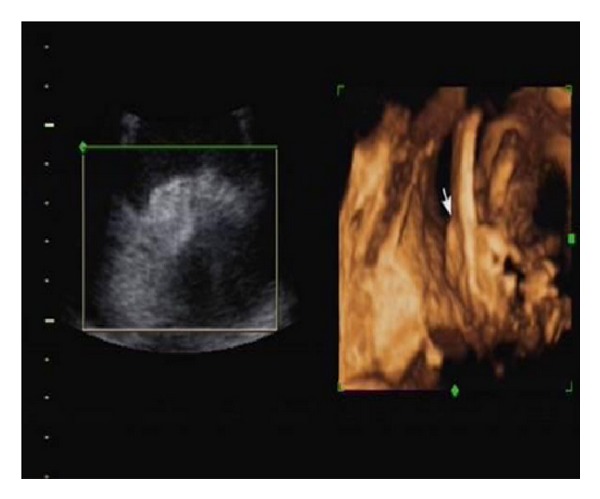
Three-dimensional sonogram of bilateral microtia.

Five cases of anomalous ears were screened out in the examination and none of them were associated with deformities of other organs. Of these one had a small bilateral microtia with malformed external auditory canal (Figures 1 and 2) and one had unilateral auricle with abnormal morphology. The remaining three cases had unilateral microtia (Figures 3 and 4).

## 4. Discussion

The development of the fetal ear is complicated; developmental disorders of the ears are not uncommon [21]. In the development course of fetal ear, changes of ear size, shape, and position may result in deformity [22, 23]. The results of our study show that size and shape of auricles and external auditory canal of aberrant fetuses can be visualized on ultrasound images including transverse-incline view at cervical vertebra level and mandible level, on parasagittal view and coronal view at external ear level, which was consistent with the findings of the literatures above. However, we did not find the difference between ear positions.

It is well known that the second trimester is the best period of prenatal ultrasonic screening for fetal malformation [4, 23–25]. The correlation analysis of auricular detection rate and gestational week in our study shows that the detection rate of two ears was negatively correlated with gestational age and was highest in 20–24 weeks which can be considered as the best period to detect external ear abnormalities. All the cases of external ear abnormalities screened out in our study were achieved in this period. In this study, five fetuses had anomalous ears; of these one had bilateral short auricles with unclearly external auditory canal and one had unilateral external with abnormal shape. The remaining three cases had unilateral microtia. Two of the 5 cases of ear abnormalities were detected where the gestational age was at 16–20 w, and the other three cases of ear abnormalities were detected at a gestational age of 20–24 w.

In the systematic examination of fetal auricles by color Doppler ultrasonography, although detection rate of ears is affected by placental amniotic fluid, the most important factor influencing the detection rate is fetal position. In the second trimester, changes in fetal movements are relatively large and fetal position is unfixed and thus bilateral external ears cannot be detected at a time. In this case, aerobic exercise during pregnancy may help the fetal movements and multiple ultrasound examinations will help locate and obtain images of two external ears.

One of the aims of the present study was to evaluate the efficacy of prenatal ultrasound screening for fetal ear abnormality. In this report, the detection rate of bilateral ears in the second trimester is highest (99.21%) in routine obstetric ultrasonography which can be used as one of the common examination items in the prenatal ultrasonography. In addition, there is no difference in detection rate of auricles between senior doctors and junior doctors.

Although fetal external ear abnormalities belong to minor physical anomalies, developmental anomalies of the external ear are still be found in some genetic diseases such as trisomy 21 and trisomy 18 [26–29]. Previous studies have confirmed that anomalous, low-set, and malformed ears are one of the common symptoms of chromosomal abnormalities; for instance, ear abnormalities are often linked with trisomy 9 (chromosome 9 abnormality), Edwards syndrome/trisomy 18 (chromosome 18 abnormality), Patau syndrome (chromosome 13 abnormality), Down syndrome (chromosome 21 abnormality), Cri du chat/chromosome 5q deletion syndrome, CHARGE (chromosome 8 abnormality), trichorhinophalangeal syndrome, Type 1 (chromosome 8 abnormality), Beckwith-Wiedemann (chromosome 11 abnormality), Jacobsen syndrome (chromosome 11 abnormality), Smith-Magenis syndrome (chromosome 17 abnormality), Emanuel syndrome (chromosome 11 and/or 22 abnormality), Turner syndrome (chromosome XO or mosaic XX/X0 abnormality), Triple X syndrome (chromosome 49 and/or XXXXX abnormality), and so forth [30] (see Table 2). These conclusions were further reinforced in our study. Among the 5 cases of ear abnormalities, 3 cases were confirmed as trisomy 21 (Down syndrome) through amniocentesis. Another case had similar ear abnormality with his grandfather. We have conducted postnatal followup studies to these participants. Those fetuses that had showed normal ears in the prenatal ultrasound screen did have normal ears after birth, with only one exception of absence of auditory canal. In those cases of fetuses where auricle of pinna were not shown in the prenatal ultrasound screen, there was one case of unilateral absence of auricle of pinna after birth, the gestational age of which was 36 + 1 w.

Therefore, ultrasound screening of external ear can be used as one of the indicators of prenatal diagnosis of fetal chromosomal abnormalities which might help in decreasing the birth defects, and ultrasound screening of the external ear should ideally be made in the period between 20 and 24 weeks of gestation.

## Figures and Tables

**Table 1:** Detection rate of fetal external ear in different gestational ages for routine obstetric ultrasonography.

Gestational age (week)	Fetus (*n*)	Number of auricles (*n*)	Detection rate (%)
16~<20	4825	9650	94.11
20~<24	7892	15784	95.19
24~<28	7256	14512	87.45
28~<32	6621	13242	77.56
32~<36	6324	12648	68.84
36~40	4245	8490	50.73

Total	37163	74326	78.98

**Table 2:** Link of ear abnormality-related syndrome with chromosomal abnormalities.

Syndromes	Chromosomal abnormality
Trisomy 9	Chromosome 9
Edwards syndrome/trisomy 18	Chromosome 18
Patau syndrome	Chromosome 13
Down syndrome	Chromosome 21
Cri du chat/chromosome 5q deletion syndrome, CHARGE	Chromosome 8
Trichorhinophalangeal syndrome, type 1	Chromosome 8
Beckwith-Wiedemann	Chromosome 11
Jacobsen syndrome	Chromosome 11
Smith-Magenis syndrome	Chromosome 17
Emanuel syndrome	Chromosome 11 and/or 22
Turner syndrome	Chromosome XO or mosaic XX/X0
Triple X syndrome	Chromosome 49 and/or XXXXX
